# Radiographic Analysis of Graft Dimensional Changes in Transcrestal Maxillary Sinus Augmentation: A Retrospective Study

**DOI:** 10.3390/ma15092964

**Published:** 2022-04-19

**Authors:** Luca Comuzzi, Margherita Tumedei, Adriano Piattelli, Gianluca Tartaglia, Massimo Del Fabbro

**Affiliations:** 1 Independent Researcher, San Vendemmiano, 31020 Conegliano, Italy; luca.comuzzi@gmail.com; 2 Department of Biomedical, Surgical and Dental Sciences, Università degli Studi di Milano, 20122 Milan, Italy; margherita.tumedei@unimi.it (M.T.); gianluca.tartaglia@unimi.it (G.T.); 3 Dental School, Saint Camillus International University for Health Sciences (Unicamillus), 00131 Rome, Italy; apiattelli51@gmail.com; 4 Dental School, University of Belgrade, 11000 Belgrade, Serbia; 5 Casa di Cura Villa Serena, 65013 Città Sant’Angelo, Italy; 6 Fondazione Villaserena per la Ricerca, 65013 Città Sant’Angelo, Italy; 7 IRCCS Fondazione Ca’Granda IRCCS Ospedale Maggiore Policlinico, 20122 Milan, Italy; 8 IRCCS Orthopedic Institute Galeazzi, Via Riccardo Galeazzi 4, 20161 Milan, Italy

**Keywords:** biomaterials, maxillary sinus, sinus augmentation, xenograft, transcrestal procedure

## Abstract

Background. The maxillary sinus lift is a popular and predictable technique associated with implant-supported rehabilitation of the severely atrophic maxilla. The aim of the present retrospective study was to investigate the effectiveness of transcrestal maxillary sinus augmentation and the graft resorption pattern using different heterologous bone substitutes. Methods. A total of 75 sinus-grafting procedures were performed and 89 implants were placed in 66 patients, 24 males and 42 females, with mean age 67.9 ± 10.64 years (range 43–84 years). Nineteen subjects were smokers. The mean follow-up period was 93.33 ± 54.71 months (range 14–240 months). Clinical and radiographical evaluations were performed. Graft height and width were measured at baseline and at the latest follow-up. Results. Mesiodistal and vertical resorption averaged 9.3 ± 20.7% (standard deviation), and 5.04 ± 9.9% of the postoperative size, respectively, considering the graft as the unit. Linear regression analysis showed that graft resorption in both the vertical and the mesiodistal dimension is independent of the follow-up time. Conversely, there was a trend for greater resorption when increasing the postoperative graft size, in both vertical (*p* = 0.001) and horizontal (*p* = 0.007) dimensions. When grouping the dimensional changes by graft particle size (only small (<300 μm) particles, combination of small and medium (>500 μm)/large (>1000 μm) particles, and only medium/large particles), there was a trend for greater resorption associated with smaller particles, but it was not significant; neither in the mesiodistal nor in the vertical dimension (*p* = 0.17 and *p* = 0.25, respectively). No implant was lost during the observation period. In conclusion, the transcrestal technique for maxillary sinus augmentation documented a high level of predictability. The low clinical morbidity and the contextual dental implant positioning is clinically useful in relation to a significant reduction of the time required for implant restoration, a consistent decrease of the number of surgical phases, and a cost-effectiveness approach for the rehabilitation. The graft resorption pattern in all cases was compatible with persistent implant protection and support.

## 1. Introduction

Posterior maxillary atrophies represent a critical occurrence in dental implant procedure and a limitation for a fixed rehabilitation of this area [1,2]. This evidence is generally associated with physiological factors and age, but it is also correlated in the literature to teeth loss, infections, passive prosthetic loading, trauma, cysts, and neoplasms [3,4]. In presence of an optimal vertical distance between the maxillary and mandibular occlusal arches, the most common regenerative approach is the sinus augmentation procedure, which represents a highly predictive technique to recreate the correct vertical length for the dental implant positioning [5,6,7]. As described by Tatum et al., this surgical technique consists of the elevation of the Schneiderian membrane, which covers the internal cavity of the antrum, and the positioning of a bone graft [8]. The rationale behind this technique lies in the making of a favorable regenerative space for blood clot stability in order to produce new bone formation at the level of the grafted site [8,9,10]. The surgical approach can be performed through a lateral antrostomy in order to produce an access to the sinus cavity and bone grafting [8,11,12]. This technique takes advantage of increased visibility and manageability of the surgical site in order to produce an adequate membrane elevation and maintain an opportune three-dimensionality of the graft [13,14]. On the contrary, a transcrestal approach can reduce surgical morbidity because of the avoidance of lateral osteotomy and a more localized sinus floor elevation. The transcrestal approach often allows for an immediate dental implant positioning, avoiding a second surgical step [10,15,16]. In addition, the rate of sinus membrane perforation, the most frequent intraoperative complication of sinus augmentation procedure, was reported to be lower with the transcrestal approach as compared to the lateral approach [17]. However, since the former is considered a “blind” technique, it is possible that some perforations remain undetected and their true incidence is greater than that reported. Many different transcrestal techniques, using a variety of instruments, have been described in the literature, i.e., manual Summers osteotomes [18,19,20], calibrated osseodensifying dental drilling [21], and fluid and/or hydrodynamic devices [22,23,24,25]. Many studies reported that the implant apical site in contact with the graft is subjected to a continuous remodeling and shrinkage process that could be correlated to a functional adaptation to the loading [26,27]. This aspect is also accompanied with a centrifugal resorption that follows the antrum pneumatization vector that could influence the graft resorption pattern over time [28]. In this way, many authors reported a higher volume stability of synthetic bone substitutes compared to the autogenous bone graft [15,29,30,31,32], despite the latter representing the optimal biomaterial for bone regeneration due to its osteoconductive, osteoinductive, and osteogenic properties [33,34]. Moreover, sinus anatomy could also influence the graft remodeling pattern. For example, a narrow antrum cavity with a reduced bucco-palatal distance could support a more effective new bone formation process, but have a higher exposure to surgical complications and membrane perforation, than wider antrum cavities [35]. On the contrary, the latter are disadvantageous for blood clot stabilization and less supportive for the grafted site [35]. At this scope, Scarano et al. reported that the mechanical properties of the particulated scaffold could be improved through the colligative properties of platelet-derived hemocomponents that take advantage of a high surgical plasticity and the biological action of the super-concentration of the autologous growth factor of the sticky graft [36]. The aim of the present retrospective investigation was to evaluate the bone graft stability of maxillary sinus floor augmentation via transcrestal approach, considering the type and size of the biomaterials’ particles.

## 2. Materials and Methods

### 2.1. Study Design

This retrospective study was conducted in a private dental practice in Conegliano (TV) Italy. The study was in accordance with the Good Clinical Practice guidelines and followed the guidelines of the Declaration of Helsinki. Ethical Board approval was not required, given the observational retrospective nature of the study, which was conducted in a private clinic, under daily clinical practice conditions, following standard protocols. Informed consent for data evaluation and publishing has been obtained from all included subjects.

All patients included signed a written consent form before the intervention. The present investigation enrolled patients who required implant rehabilitation of the posterior maxilla and a maxillary sinus augmentation procedure with the transcrestal technique using a heterologous bone substitute as grafting material, and who received follow-up at least 3 months after prosthesis delivery.

### 2.2. Surgical Procedure

Surgeries were performed by a single surgeon. Patients were treated from January 2001 until September 2020. The surgery consisted of one-stage implant placement and sinus augmentation via trancrestal technique. All patients received a panoramic radiograph and a Cone Beam Computed Tomography scan before surgery. In all subjects, local anaesthesia was performed with 4% articaine and 1:100,000 epinephrine (Pierrel, Milan, Italy) before the surgery.

An intracrestal incision was made on the medial side and continued to the distal side, and a full-thickness flap was raised to gain access to crestal bone.

For the implant site preparation, a lance-shaped drill of the implant kit was used, followed by a 2 mm drill, until reaching the internal cortical of the maxillary sinus. It was important to maintain a sub-preparation of the site in accordance with the drilling protocol (generally 3 mm) of the implant company always used below the internal cortex of the sinus.

The internal sinus cortical was not fractured with the osteotome but rather depleted with internal irrigation with a particular blunt tip capable of gradually consuming the bone without fracturing it. (Guided Sinus Lift Drill kit Dr. Cosci Ferdinando, Isomed Srl, Albignasego (Padua), Italy).

Once the cortical layer was removed, the sinus membrane was gently detached, and the augmentation was performed with the addition of particulate bone substitute (from 0.2 cc to 0.5 cc per site).

The dental implants were inserted at 30 N/cm to the crestal level and the healing screws, of 3–5 mm height, were directly applied in order to make them monophasic. Vertical U sutures were made, mesial and distal to each implant. Antibiotic prophylaxis with Augmentin 1 g 2 times a day for 6 days and painkillers if needed were prescribed. Sutures were removed 10 days after surgery.

Check-up was conducted at 1 month after surgery and radiography after 3 months. At approximately 4–6 months, definitive impressions were taken to complete the cases over the next 2 months.

### 2.3. Biomaterials

In the present study, all the biomaterials used for the bone sinus augmentation were produced by Tecnoss^®^ (Giaveno, Italy)

OsteoBiol^®^ mp3^®^ is a cortico-cancellous porcine bone mix, composed of 90% granulated mix and 10% collagen gel. Two types of granulometry are available for OsteoBiol^®^ mp3^®^: 600–1000 µm and 1000–2000 µm.

OsteoBiol^®^ Putty is a cortico-cancellous porcine bone mix, composed of 80% granulated mix and 20% collagen gel. This material has a plastic consistency due to the presence of collagen gel loaded with 80% micronized bone mix and has a granulometry up to 300 µm.

OsteoBiol^®^ Gel 40 is another cortico-cancellous heterologous bone mix. It is made of a collagen matrix (type I and III), obtained using an exclusive Tecnoss^®^ process, loaded to 60% of its volume with micronized porcine bone granules (granulometry up to 300 µm).

OsteoBiol^®^ GTO^®^ is a heterologous bone grafting material made of a mix of collagenated cortico-cancellous granules of size ranging from 600 to 1000 µm. OsteoBiol^®^ GTO^®^ is composed of 80% granulated mix of porcine origin and 20% OsteoBiol^®^ TSV Gel.

OsteoBiol^®^ Apatos^®^ is a cortico-cancellous porcine bone mix with a granulometry of 600–1000 µm and 1000–2000 µm. The tissue collagen is degraded and physically appears as radiopaque granules of mineral hydroxyapatite.

Bioresorb is a synthetic material composed of ß tricalcium phosphate (Ca)_3_(PO_4_)_2_, with granulometry of 500–1000 µm, generally used in association with OsteoBiol^®^ Putty or autogenous bone.

Autologous bone was taken from the drill used to prepare the implant site.

### 2.4. Radiographic Evaluation

All patients underwent the same pre- and post-surgical protocol. Intraoral radiographs taken with the parallel technique were used for the measurements and were analyzed at the time of surgery and at the longest follow-up by a single experienced evaluator.

Using the Libre Office program (https://it.libreoffice.org/, accessed on 23 January 2021), the evaluator underwent a calibration procedure, consisting in measuring the set distances on a sample of 10 radiographs. The implant length and width were used as reference for calibration. An error was considered acceptable when the maximum difference between consecutive measurements of the same distance was below 5%.

The following distances were measured at insertion (baseline) and at follow up:

Graft width (mesiodistal extension): the distance from the most mesial point of contact of the graft with the sinus floor, to the most distal one (at graft level).

Mesial and distal extension: the distance from the most mesial/distal point of contact of the graft with the sinus floor to the mesial/distal aspect of the (most mesial/distal) implant (at graft level).

Vertical implant apex-graft: the distance from the central point of the apical end of the implant to the most apical point of the graft, along the implant axis (at implant level).

Total vertical bone height: the distance from the bone crest to the most apical point of the graft, along the implant axis (at implant level).

Residual bone height at the implant site: height of the sinus floor, expressed as the mean of the values measured at the mesial and distal aspect of each implant (at implant level).

**Figure 1:**
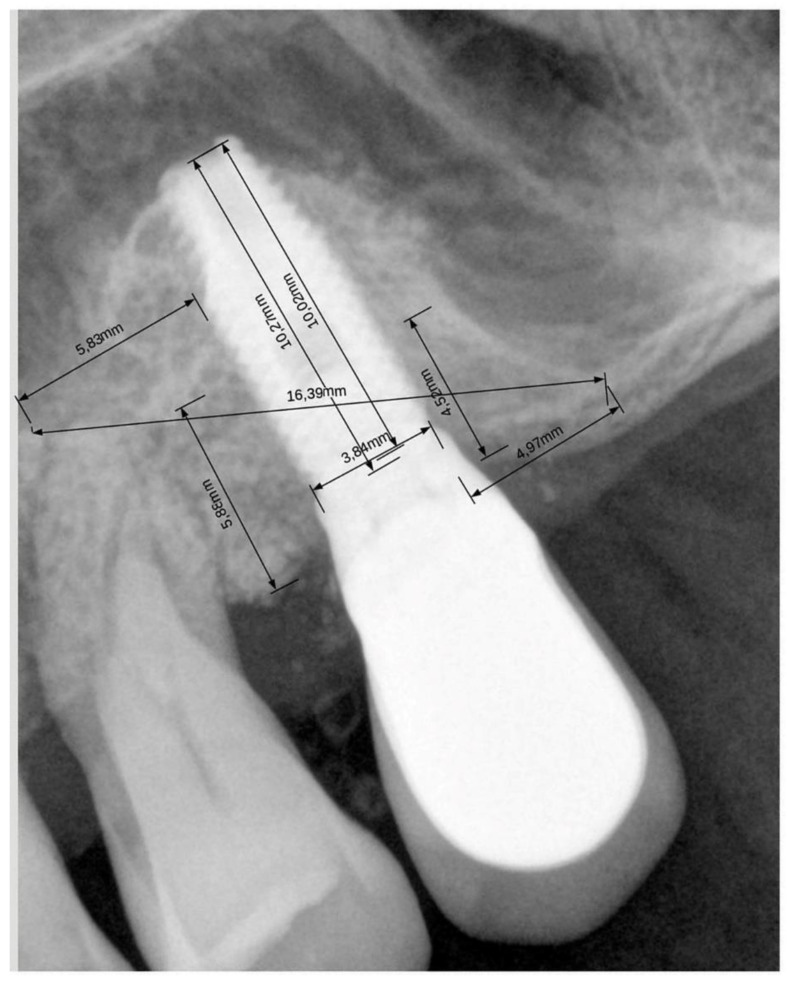
An example of the measurements taken at each implant is shown. In this case, mesiodistal graft width was 16.39 mm, distal extension was 4.97 mm, mesial extension was 5.83 mm, residual bone height was 5.2 mm ((5.88 + 4.52 mm)/2), total vertical bone height was 10.27 mm, implant length and diameter were 10 mm and 3.8 mm, respectively. Due to a mistake, the unit on the image was set in “cm” instead of “mm”.

For calibration of each image, the known implant length and diameter were used as reference. Figure 1 shows an example of the measurements.

The change between baseline and follow-up measurement was calculated for each distance.

### 2.5. Statistical Analysis

The software Graphpad Prism version 5.1 was used for statistical analyses. The significance was set at *p* = 0.05. Descriptive statistics was used to describe the features of the sample, using mean values and standard deviations (SD). The difference between baseline and follow-up values was evaluated using paired Student *t*-test in case of normal distributions. Normality of the distributions was assessed using the D’Agostino and Pearson’s omnibus normality test. Linear regression analysis between baseline values and changes (both in mm and in percentage), and between changes and follow-up time, was undertaken. One-way analysis of variance (ANOVA) was used to compare graft dimensional changes among the various grafting biomaterials. For this analysis, the biomaterials were aggregated into 3 groups, according to the granule size: <300 μm (only injectable grafts: Putty and Gel 40), combined grafts (Putty and Gel 40 combined with other materials with granulometry > 500 μm), >500 μm (grafts not including Putty and Gel 40). The difference between groups was evaluated using the Tukey test. Values of vertical change from adjacent implants in the same patients were averaged. The level of significance was set at *p* = 0.05.

## 3. Results

Sixty-six patients were enrolled in the present study. Nine of them had a bilateral sinus augmentation, so that a total of 75 sinus grafting procedures were performed. Twenty-four were males and forty-two were females, with a mean age at surgery of 68 ± 10.6 (standard deviation, min 43/max 84) years. A total of 89 implants were inserted in the posterior maxilla.

Forty-seven patients were non-smokers, 9 smoked 5 cig/day, 1 patient 8 cig/day, 7 patients 10 cig/day and 2 patients smoked 15 cig/day (Table 1). The follow-up averaged 93.3 ± 54.7 months (min 14/max 240 months).

Different bone substitute materials were used for maxillary sinus augmentation (Table 1). Patients undergoing bilateral sinus augmentation received the same biomaterial in both sides. Considering the graft as the unit, mesiodistal and vertical resorption at the latest follow-up averaged 9.3 ± 20.7% and 5 ± 9.9% of the postoperative size, respectively.

Table 2 describes the implant distribution according to diameter and length, while Table 3 describes the implant distribution according to the maxillary site. Of the 89 implants inserted, the results of only 86 were considered because three implants had incomplete data regarding graft dimension change.

Table 4 describes the main results of the dimensional changes of the graft. All the data are presented in mm as mean values and standard deviations, except for changes that are expressed as percentages, after normalization of the actual change in mm by the baseline distance. Overall, the grafting material resorbed significantly in both mesiodistal and corono-apical dimension.

### 3.1. Regression Analysis

**Figure 2:**
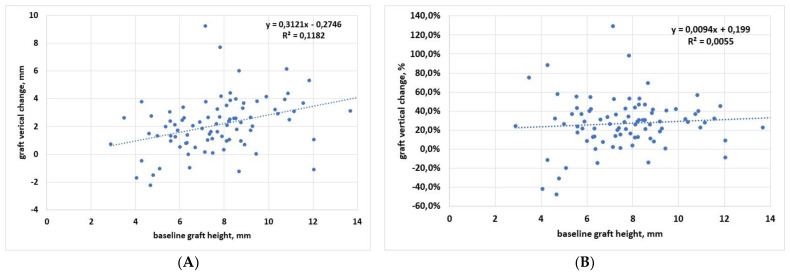
Regression analysis for vertical dimension change with respect to baseline. (**A**) Data in mm; (**B**) data in percentage.

Figure 2 shows regression analysis of the vertical dimension, in which graft height change, expressed both in mm (Figure 2A) and in % (Figure 2B), was described in correlation with baseline graft height. Linear regression analysis showed that there was a trend towards an increase of the graft height change when the initial graft height increases. The slope was significantly different from zero when the analysis was run for data expressed in mm (*p* = 0.001), while despite a similar trend, it was not significantly different from zero when considering the percentage data (*p* = 0.49).

When vertical graft changes were correlated to follow-up duration, linear regression analysis showed that the slope was not significantly different from zero, both when considering the data in mm (*p* = 0.09) and in percentage (*p* = 0.18). This indicates that graft resorption in the vertical dimension is independent of the follow-up time.

**Figure 3:**
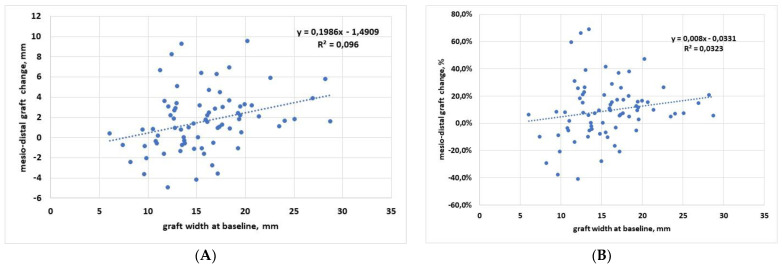
Regression analysis for mesiodistal dimension change respect to baseline. (**A**) Data in mm; (**B**) data in percentage.

Figure 3 shows regression analysis of the mesiodistal dimension, in which graft width change, expressed both in mm (Figure 3A) and in % (Figure 3B), was described in correlation with baseline graft width. Linear regression analysis showed that there was a trend towards an increase of the graft width change when the initial graft width increases. The slope was significantly different from zero when the analysis was run for data expressed in mm (*p* = 0.007), while despite a similar trend, it was not significantly different from zero when considering the percentage data (*p* = 0.12).

When mesiodistal graft changes were correlated to follow-up duration, linear regression analysis showed that the slope was not significantly different from zero, both considering the data in mm (*p* = 0.23) and in percentage (*p* = 0.47). This indicates that bone remodeling produces a significant dimensional reduction of the biomaterial in the mesiodistal dimension, which is independent of the follow-up time.

### 3.2. Effect of Material

**Figure 4:**
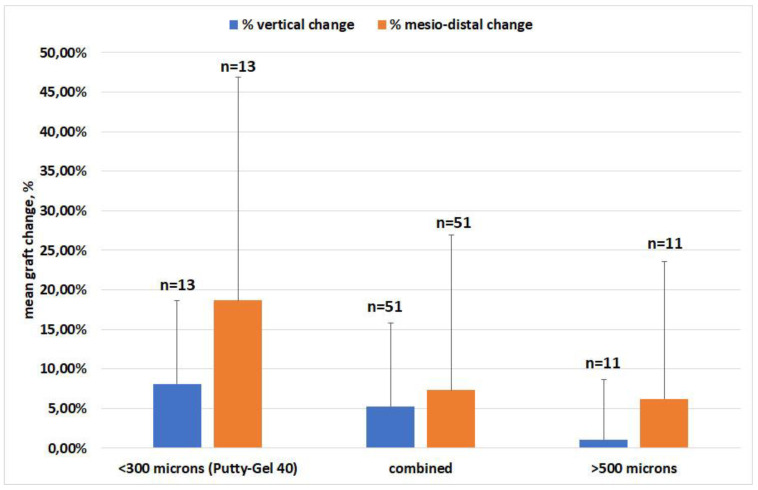
Mean vertical and mesiodistal changes, with standard deviations, for the three groups of grafting biomaterials. The biomaterials are grouped according to the granule size, as described in the text. Data are expressed in percentage of change, respective to postoperative values. The graft was the unit of analysis. The number of cases per groups is also indicated.

The analysis of graft resorption in both mesiodistal and vertical dimensions according to the graft material used is synthesized in Figure 4. The variations are expressed as percentages. All 75 grafts could be used for the analysis. ANOVA showed that there was no significant difference in dimensional change among the various groups of materials, neither in the mesiodistal (*p* = 0.17) nor in the vertical (*p* = 0.25) dimensions. Despite an apparent trend towards larger changes when reducing the granulometry of the grafts, both in the vertical and mesiodistal dimensions, there was no significant difference between groups, due to large within-group variability.

## 4. Discussion

This study confirmed good performance from the sinus augmentation procedure under daily practice conditions, using different grafting materials of various origin (mostly heterologous of porcine origin, and in a few cases synthetic and autogenous). The same operator treated all patients, and the same evaluator performed all measurements, thereby minimizing a quote of the variability of results. The present study found that graft shrinkage in both horizontal and vertical dimensions is independent of the follow-up duration and irrespective of the graft material used. The first finding would imply that, once the graft remodeling occurs, the graft size tends to stabilize, showing minimal changes over time. The mean change in the mesiodistal and in the vertical dimensions amounted to about 10% and 5% of the graft size at placement. The mean graft height apical to implant apex averaged 1.8 mm at insertion and 1.06 mm after a mean of 82.3 months follow-up, thereby warranting long-term apical protection in the large majority of cases. A recent multicenter study on graft remodeling in transcrestal sinus floor elevation reported a median graft height apical to implant apex of 1.4 mm after 6–12 months follow-up (comparable to that observed in the present study), and a tendency to decrease at later follow-up intervals [37]. That paper, however, did not assess if such tendency was significant or not, and did not report the magnitude of the decrease in the long term. Indeed, in our study, the slope of the linear regression between vertical change and follow-up duration was also negative, but not significantly different from zero (Figure 3).

The dimensional change of the bone graft around implants after maxillary sinus augmentation is a longstanding matter of investigation. In fact, graft stability may represent an important prognostic factor for implant success in the long term [38,39]. Several clinical and radiographic studies have evaluated graft height and volume changes in maxillary sinuses augmented with various types of grafting materials [39,40,41,42,43,44]. While autogenous bone grafts were found to have unpredictable resorption [45,46,47], other common bone substitutes such as deproteinized bovine bone matrix demonstrated extremely slow or no resorption at all after several years [48,49]. If bone substitutes do not undergo remodeling, they will not be replaced by new bone. As a consequence, they might retain biological and mechanical characteristics different from those of the surrounding native or newly formed bone [50]. In particular, they might lack a well-structured microvascular bed, typical of mature bone, which is of utmost importance in the immune defense against possible infections [51,52]. During graft healing, a given reduction of graft dimension is a physiological consequence of early remodeling, and only small changes should occur thereafter [39].

The fact that no significant difference in remodeling was found among the various groups of biomaterials was partly related to the high variability in outcomes and partly to the fact that some grafting materials were used in a small number of cases, as can be seen in Table 1. In particular, the materials with small granulometry, OsteoBiol^®^ Putty and OsteoBiol^®^ Gel 40, were used alone in only 10 patients (13 grafts), while they were more frequently used in combination with other materials with greater particle size (e.g., Bioresorb, OsteoBiol^®^ GTO^®^, OsteoBiol^®^ Apatos^®^, OsteoBiol^®^ mp3^®^). OsteoBiol^®^ Putty and OsteoBiol^®^ Gel 40 used alone showed higher resorption in both dimensions, though not significantly, as compared to the other groups. This would suggest that, based on the present findings, OsteoBiol^®^ Putty and OsteoBiol^®^ Gel 40 might achieve better results when used in combination with other materials to preserve the graft size. Of course, due to the heterogeneity among different groups of materials and small sample sizes, this interpretation might be taken with caution. A few studies have investigated the effect of particle size on the graft healing outcomes; some reported better radiographic and histomorphometric results (in terms of newly formed bone, vascularization, and bone formation markers expression) for the grafts with larger particles [53,54], while one other found no significant differences [55]. However, these studies are hardly comparable to the present study because they refer to the lateral approach and mostly evaluated deproteinized bovine bone.

Another interesting finding is that in both vertical and horizontal dimensions, larger changes were associated with larger grafts (the linear regression slope was positive and significant in both cases, considering the changes in mm (Figure 2A and Figure 3A). Greater vertical resorption with higher grafts is not an unexpected finding. A recent study on graft regeneration, based on histomorphometric analysis, reported that new bone formation follows a gradient from native bone (the original sinus floor) towards the most apical part of the augmentation region [56,57]. They observed that the greater the distance from the sinus floor, which constitutes the main source of precursor cells, oxygen, and nutrients for the graft, the lower the amount and quality of newly formed bone in the graft. Hence, the most apical parts of the graft will have a greater risk of resorption, and the risk will increase in the highest grafts. A recent systematic review of RCTs investigated the effect of residual bone height and vertical graft size on graft shrinkage, and found analogous results [58]. In particular, this review suggested that minimizing the augmentation volume might have favorable consequences for graft healing and stability, especially when using alloplasts and xenografts. Different considerations can be applied to the trend for higher resorption observed in wider grafts in the mesiodistal dimension. The larger (and the higher) the graft, the greater the perturbation of the sinus physiology, especially in the transcrestal approach, which involves more restricted regions than the lateral approach. It can be hypothesized that, as a reaction to an initial stretch of the sinus membrane and sinus space reduction, an adaptation of the graft volume follows with resorption of the peripheral regions, which in turn allows sinus space expansion, until a new sinus volume is reached. Of course, these observations need to be verified by dedicated studies with a wider sample size, while also considering that the regression slope in both horizontal and vertical dimensions was not significantly different from zero when the change was expressed in % (Figure 2B and Figure 3B). One limitation of the study is that all measurements were based on 2D radiographs. Of course, 3D reconstructions can give a more precise picture of the graft extension and the remodeling pattern. However, multiple assessments of the patients with computed tomography remain subject to ethical restrictions. Furthermore, in the sample size of patients considered, some of which having been treated more than 15 years ago, only periapical radiographs were available at follow-up.

Another limitation of the present retrospective study was represented by the low number of samples for the different biomaterials used, which prevents a sound comparison of their performance. In fact, only for three types of graft were there at least ten samples; these included OsteoBiol^®^ Putty in combination with Bioresorb, autogenous bone, or OsteoBiol^®^ mp3^®^. Results obtained with OsteoBiol^®^ GTO^®^ are promising, but follow-up was relatively short, and long-term studies are necessary to validate its effectiveness.

In conclusion, it was observed that all materials used in the present study for transcrestal maxillary sinus augmentation undergo a resorption pattern that depends on the baseline dimension of the graft—which could be related to the graft particle size—but were in all cases compatible with persistent implant protection and support.

## Figures and Tables

**Table 1:** Features of the patient sample and distribution of biomaterials used.

Gender	24 Males/42 Females
Age, years	67.9 ± 10.6 (range 43 to 84)
Smoking habits	47 nonsmokers
	9 patients 5 cig/day
	1 patient 8 cig/day
	7 patients 10 cig/day
	2 patients 15 cig/day
Follow-up, months	82.3 ± 54.7 (range 14 to 240)
Grafting material	11 patients: OsteoBiol^®^ mp3^®^ + OsteoBiol^®^ Gel 40
	7 patients: OsteoBiol^®^ Putty + Bioresorb
	13 patients: OsteoBiol^®^ Putty + autogenous bone
	3 patients: OsteoBiol^®^ Putty + Bioresorb + autogenous bone
	7 patients: OsteoBiol^®^ Putty
	8 patients: OsteoBiol^®^ Gel 40 + autogenous bone
	3 patients: OsteoBiol^®^ Putty + OsteoBiol^®^ Gel 40
	6 patients: OsteoBiol^®^ GTO^®^
	2 patients: OsteoBiol^®^ Apatos^®^ + OsteoBiol^®^ Gel 40 + autogenous bone
	1 patient: autogenous bone + Bioresorb
	2 patients: OsteoBiol^®^ Gel 40 + OsteoBiol^®^ Apatos^®^
	4 patients: OsteoBiol^®^ GTO^®^ + autogenous bone
	3 patients: OsteoBiol^®^ Gel 40 + Bioresorb

**Table 2:** Implant distribution according to the diameter and length.

Length, mm	Diameter, mm									Total
	3.5	3.75	3.8	4	4.1	4.2	4.3	4.5	5.0	
8.5	-	-	-	1	-	-	-	-	-	1
9.0	-	-	-	-	-	-	-	1	9	10
10.0	1	-	4	3	2	1	5	15	1	32
11.0	-	-	1	6	-	-	-	10	8	25
11.5	1	-	-	7	-	2	1	4	-	15
13	-	1	-	3	1	-	-	1	-	6
Total	2	1	5	20	3	3	6	31	18	89

**Table 3:** Implant distribution per location in the maxilla.

Implant Site	Number of Implants
Right first premolar	2
Right second premolar	11
Right first molar	31
Right second molar	11
Left first premolar	1
Left second premolar	5
Left first molar	20
Left second molar	8
Total	89

**Table 4:** Dimensional measurements of the graft, changes, and significance.

Distance Measured	Baseline Mean ± SD (Range), mm	Follow-Up Mean ± SD (Range), mm	Change Mean ± SD (Range), %	*p*-Value	Total Number	Unit
Mesiodistal graft width	15.7 ± 4.6 (6.0, 28.7)	14.1 ± 4.67 (4.0, 27.1)	9.3% ± 20.7% (−40.9%, 68.8%)	*p* < 0.001	75	Graft
Mesial extension	4.3 ± 2.1 (0, 9.5)	3.7 ± 2.2 (0, 9.6)	13.8% ± 38.1% (−74.6%, 100%)	*p* < 0.001	75	Graft
Distal extension	5.0 ± 2.4 (0.0, 14.3)	4.2 ± 2.3 (0, 9.5)	8.8% ± 46.5% (−126.6%,100%)	*p* = 0.003	75	Graft
Vertical implant apex-graft	1.8 ± 1.54 (−2.1, 7.6)	1.0 ± 1.6 (−4.7, 7.5)	41.5% ± 85.5% (−312.0%, 100%)	*p* < 0.001	86	Implant
Total vertical bone height	13.4 ± 1.9 (8.7, 19.1)	12.6 ± 2.1 (6.1, 18.9)	5.3% ± 9.74% (−23.0%, 31.5%)	*p* < 0.001	86	implant

## Data Availability

The authors are available to share the data upon request.
